# Environmental Drivers of Local Demography and Size Plasticity in Fire Salamanders (*Salamandra salamandra*)

**DOI:** 10.3390/ani14192869

**Published:** 2024-10-05

**Authors:** Ulrich Sinsch

**Affiliations:** Department of Biology, Zoology Group, University of Koblenz, 56070 Koblenz, Germany; sinsch@uni-koblenz.de

**Keywords:** skeletochronological age estimation, longevity, growth rate, habitat quality, landscape resistance, heavy metal contamination

## Abstract

**Simple Summary:**

Age and size variation in widespread amphibians are often related to latitudinal or altitudinal temperature gradients. Species with toxic skin secretions, such as the fire salamander *Salamandra salamandra*, are an exception to the rule because their survival rates are relatively unaffected by predation, the major source of amphibian mortality. In contrast, average adult size varies greatly among populations, but variation is unrelated to geographical gradients. This study on four neighboring fire salamander populations inhabiting the catchments of low-order streams in the upper middle Rhine Valley (Koblenz, Germany) focuses on the identification of local environmental drivers of variation in age and body size distributions. I collected 192 individuals at two localities per stream, snout–vent length measured, clipped a toe for posterior skeletochronological age determination, and released salamanders in situ again. As expected, demographic parameters were in the range of other populations with an age at maturity of 2–3 years and a maximum lifespan of 17 years, but terrestrial habitat quality accounted for 3.6% of variation among populations. Variation in adult size resulted mainly from a carry-over effect of heavy metal contamination on juvenile growth rates. In conclusion, the average adult body size is a sensitive indicator of local habitat quality.

**Abstract:**

Conspecific amphibian populations may vary widely in local demography and average body size throughout their geographical range. The environmental drivers of variation may reflect geographical gradients or local habitat quality. Among fire salamander populations (*Salamandra salamandra*), local demography shows a limited range of variation because high concentrations of skin toxins reduce mortality from predation to a minimum, whereas average adult body size varies significantly over a wide range. This study on four neighboring populations inhabiting the catchments of low-order streams in the upper middle Rhine Valley (Koblenz, Germany) focuses on the identification of local environmental drivers of variation in age and body size. I collected 192 individuals at two localities per stream, measured snout–vent length, clipped a toe for posterior skeletochronological age determination, and released salamanders in situ again. Populations were similar in age distribution. Local habitat quality accounted for a significant proportion of demographic variability, mediated by the impact of landscape-induced mortality risk, including roads and agriculture. Still, the main effect of variation in habitat quality was on adult body size, the result of growth rates of aquatic larvae and terrestrial juveniles. Larvae exposed to non-lethal heavy metal contamination in streams developed into smaller juveniles and adults than clean-water larvae, providing evidence for carry-over effects from one stage to another. The generally small average adult size in the Rhine Valley populations compared to those in other parts of the distribution range indicates the action of a still unidentified environmental driver.

## 1. Introduction

Genome and environment interact to produce phenotypes on which selection can act [1,2]. Variation in the strength and direction of these interactions influences the magnitude of phenotypic variation. Phenotypic response to environmental cues during development may vary with spatial and temporal variation in the environment and the capacity to compensate for changes in later life [3]. Lifespan is a complex quantitative life-history trait involving genetic and non-genetic factors and shows considerable variation within populations and between individuals [4]. Placing adaptive phenotypic plasticity in the framework of life history evolution, alternative phenotypes and complex life cycles can be seen as evolutionary solutions for long lifespans [5,6].

Complex life cycles like those of amphibians include a metamorphosis that links an aquatic with a terrestrial life stage and that can serve as a decoupling mechanism across a corresponding niche shift [7,8,9]. For example, carry-over effects from the aquatic into the terrestrial life stage and compensatory growth during the terrestrial stage have been found in several anuran species [10,11,12]. In caudate amphibians, spotted salamanders *Ambystoma maculatum* partially compensate for reduced growth during the larval stage with increased growth as juveniles if terrestrial habitat quality of juveniles was good [13]. Thus, conditions experienced during embryonic and larval stages may later constrain growth and survival of amphibians, affecting the longevity and body size of adults.

Stream breeding salamanders, such as *Salamandra* spp., are excellent model species to study local environmental drivers of phenotypic plasticity in aquatic and terrestrial life stages because populations inhabit small-sized catchment areas of low-order streams and individuals are rather sedentary, and therefore, gene flow among populations is frequently low to absent [14,15,16]. In the field, they can live up to 24 years (50 years in captivity; Supplementary Table S1), allowing us to detect divergent features in the realized age in response to local environmental conditions [17,18,19,20,21,22,23,24,25,26,27,28]. The potential for a relatively long lifespan is probably related to the low vulnerability to predation because the presence of toxic alkaloids is signaled by the aposematic color pattern of adults [29,30,31]. Still, aposematism does not save adults completely from being preyed on by a few snake, bird, and mammal species, and juveniles suffer from a greater number of predators [32,33,34,35,36,37,38]. Local variation of demography may also influence size distribution because salamanders grow indeterminately, but when attaining sexual maturity, the growth rate decreases sharply [17,19,20]. Consequently, the adult size mainly represents growth before sexual maturation, i.e., sensitivity to environmental influences on body size is probably greatest in the aquatic larval stage and in the terrestrial post-metamorphic juvenile stage.

This study focuses on the identification of the environmental drivers of local demography and size in the widespread fire salamander, *Salamandra salamandra.* This species experiences three distinct environments during its development: the embryonic stage intrauterine within the mother, the larval stage within slowly flowing streams, and the post-metamorphic stage in the leaf litter and wood debris of deciduous forests [34,35,36,39]. Skeletochronological age estimates in populations distributed throughout its geographical distribution from Poland in the north to Portugal and Turkey in the south (Supplementary Table S1) indicate that age at maturity varies between 2 and 4 years, while longevity ranges from 9 to 17 years [17,18,19,20,21]. Corresponding body length within a given age class may vary up to about 97 mm total length and up to about 20 mm snout–vent length. Macroecological determinants such as altitude and latitude do not explain the observed variation in age and size, which seems to be related to habitat quality [17]. If habitat quality is a major driver of demography and size variation in adult salamanders, a suitable approach to identify drivers of phenotypic plasticity is to analyze variation at local scale in populations experiencing different habitat qualities but the same macroclimate [13]. Large populations of fire salamanders inhabit almost every catchment area of small streams discharging into the upper middle Rhine Valley near Koblenz, Germany, experiencing very similar temperature and rainfall regimes [40]. Still, local habitat quality varies from nearly undisturbed in deciduous forests to heavily disturbed by human settlements, roads, mining, and agriculture. Since adult fire salamanders do not move distances exceeding a few hundred meters [14,15], this region provides ideal conditions for a small-scale approach to the environmental drivers of phenotypic plasticity.

Hypothesizing that habitat quality is a primary determinant of age and size variation, three predictions were generated and subsequently tested in this study: (1) poor stream water quality (heavy metal contamination, eutrophication) reduces the size at metamorphosis; (2) juveniles emerging from polluted streams will grow to small-sized adults independent of the quality of the terrestrial habitat; and (3) local demography reflects the terrestrial habitat quality.

## 2. Materials and Methods

### 2.1. Study Area

**Figure 1:**
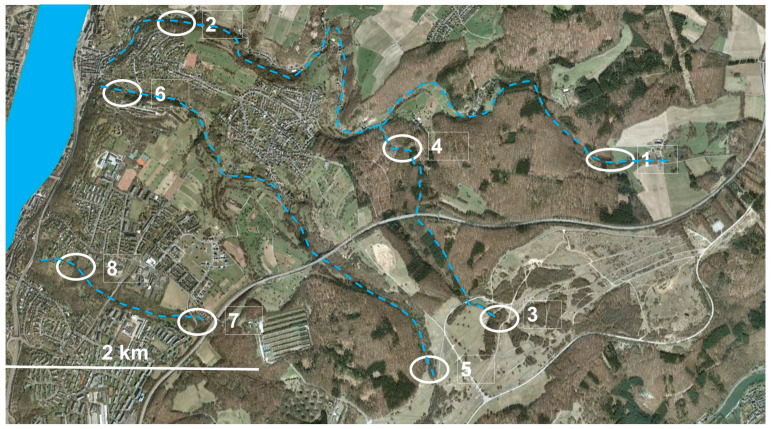
Aerial view on the study area in January 2010. The four low-order streams are indicated by the blue broken lines (the cased sections are not shown), the eight sampling areas inhabited by *Salamandra salamandra* by white ellipsoids. The urban areas are part of the city of Koblenz, east of the Rhine River (blue area). Details on the streams (Mühlenbach 1 and 2, Wintersbornbach 3 and 4, Griesentalbach 5 and 6, and Bienhornbach 7 and 8) and hydrography are given in the text. Source: Google Earth, modified.

The study area pertains to the municipal area of Koblenz (Rhineland-Palatinate, Germany) east of the Rhine River. It includes the hydrographic systems of four streams, all of which are inhabited by *Salamandra salamandra* (from north to south): Mühlenbach, Wintersbornbach, Griesentalbach, and Bienhornbach. The Winterbornbach discharges into the Mühlenbach, which discharges directly into the Rhine River as do the other two streams. The sampling areas near the headwaters are labeled 1, 3, 5, and 7, those near the stream mouth are 2, 4, 6, and 8 (Figure 1). Most areas were sections of densely vegetated dells with shrubs and deciduous forest, but some also included current and former vineyards fortified with dry stone walls, dirt roads, and paved lanes.

The Mühlenbach is the longest stream, with a length of 6.9 km and draining an area of 961 ha. The headwaters originate near the farm Mühlenbacher Hof, and the stream discharges cased in the urban district of Ehrenbreitstein. Sample area 1 was at 252–288 m asl, area 2 at 92–116 m asl about, 0.8 km distant from the stream mouth. During 1847–1960, the mine Grube Mühlenbach located in the catchment area of the Mühlenbach extracted ore containing Zn and Pb. The Mühlenbach flows through partially contaminated mine waste. Slopes near the stream mouth are used as vineyards for more than 150 years. In the local viniculture, several types of pesticides have been applied in the past, among them flazasulforon, glyphosate, and copper solutions [41]. The entry and toxicity of organic pesticides and copper in vineyard streams have been documented for Rhineland–Palatinate [42,43]. The Wintersbornbach is the shortest stream with a length of 1.9 km and a drainage area of 169 ha. The headwaters are within the former military training area Schmidtenhöhe (sample area 3 at 254–282 m asl) but unlike the other streams, the Wintersbornbach discharges at 167 m asl into the Mühlenbach near Korn’s Mühle (sampling area 4 at 196–212 m asl). The Griesentalbach with 4.5 km is the second-longest of the study area and drains 378 ha. The headwaters are also located in the Schmidtenhöhe (sampling area 5 at 276–288 m asl), and discharges cased together with the Mühlenbach in Ehrenbreitstein. At about 0.5 km distant from the stream mouth sampling area 6, it was at 98–118 m asl. The Bienhornbach has a length of 2.4 km and drains an area of 186 ha discharging cased in the urban district of Pfaffendorf [40]. Sampling area 7 at 188–203 m asl was near the headwaters and area 8 was at 144–160 m asl at a distance of about 0.6 km from the stream mouth.

### 2.2. Field Sampling

Adult salamanders were sampled by hand within forested areas or dirt roads or paved lanes during autumn (9 September–17 October 2010). Permission for the collection of salamanders was granted by the Struktur- und Genehmigungsdirektion Nord (Koblenz, Germany). After sunset, salamanders encountered by chance foraging were collected between 2000 and 2200 h using visual encounter transects parallel to the stream at each of the eight sampling localities (Figure 1). Independent of the length of the transect and time spent, local sampling ended when 10 individuals of each sex had been collected. In most sites, sampling included several nights. The exact sampling site of each individual (coordinates, altitude above sea level) was recorded using a Garmin GPS. Each individual was sexed by direct observation of the shape of the cloaca. Snout–vent length (SVL) was measured to the nearest 1 mm using a caliper [25,40]. The longest digit (third or fourth toe) of the right hind limb was removed with an alcohol-cleaned surgical scissor and stored in 4% formaldehyde for skeletochronological analysis. The toe-clipping procedure was approved by the ethical committee of FB3, University of Koblenz-Landau (# Si 2010/01). Following disinfection of the wound with alcohol, each individual was released at the point of capture. Individuals were considered mature if they exhibited external sexual characters. In females, cloacal lips are poorly developed and flattened, whereas in males, the cloaca is well developed, large, and convex, and the cloacal lips form a parted gap [14]. To establish von Bertalanffy growth curves, some juveniles (small individuals without obvious external sexual characters) were collected along with the adults. The growth curves were rooted with recently metamorphosed individuals, which were collected at each sampling site (n = 3 per site) during the summer of 2011. SVL was measured and a toe clipped analogous to the procedure applied to adults.

### 2.3. Skeletochronological Age Estimation

**Figure 2:**
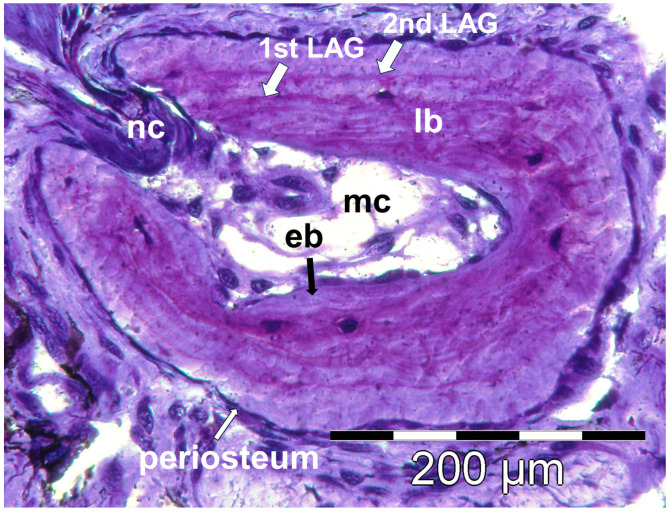
Histological interpretation of a cross section at the diaphysis of the second phalange bone of a female (SVL 71 mm, 2 LAGs, sampling site 2). Abbreviations: eb = endosteal bone; LAG = line of arrested growth; lb = lamellar bone; mc = medullary cavity; nc = nutrition canal.

Laboratory protocols followed the standard methods of skeletochronology [24,44]. The complete samples (bones and attached soft tissue) were embedded in HistoresinTM (JUNG) and stained with 0.5% cresylviolet. Preferentially, the diaphysis of the second phalange of each sample was cross-sectioned at 12 µm using a JUNG RM2055 rotation microtome. Cross sections were examined using light microscopy for the presence of growth marks in the periosteal bone at magnifications of 400× using an OLYMPUS BX 50 (Tokyo, Japan). Four types of growth marks were distinguished in the periosteal bone [44,45]: (1) strongly stained single lines of arrested growth (LAGs), separated by faintly stained broad growth zones; (2) groups of two or more strongly stained thin lines of arrested growth (multiple LAGs), separated by faintly stained narrow growth zones and followed by a broader growth zone; (3) zones of fast bone growth alternating with stronger stained lamellae of reduced growth within the first broad growth zone produced in the period between metamorphosis and first hibernation; (4) faintly stained growth zone between the outermost LAG and the inner cellular layer of the periosteum identified by its flat nuclei (Figure 2). I selected diaphysis sections in which the size of the medullar cavity was at its minimum and that of periosteal bone at its maximum. The number of LAGs was assessed to estimate age as the number of completed annual cycles. Please note that an individual with 3 LAGs was in his fourth year of life, having completed three years and almost entirely the fourth growth period from spring to autumn in the year of sampling.

### 2.4. Potential Drivers of Local Mortality and Size Variation

Longevity and SVL variation in salamander populations may be affected by local aquatic and terrestrial habitat quality. Adults migrate 35–375 m per night (average 127 m) and disperse up to 980 m [15,46]. Therefore, habitat quality within a radius of 100 m of the sampling locality is representative for most of the area used for foraging and that within a radius of 500 m for dispersing behavior. Four types of potential environmental drivers of demography and size plasticity were subsequently evaluated: (1) temperature; (2) heavy metal pollution and eutrophication of streams; (3) landscape-induced risk of mortality (LIMR); (4) landscape resistance to migration (LRM).

The headwaters of the studied streams are located about 220 m above the level of the Rhine River. The altitudinal difference between headwater area and stream mouth translates into an air temperature difference of 2.1 °C as indicated by the long-term average of 11.8 °C at 68 m asl (DWD station Neuwied) and of 9.7 °C at 265 m asl (DWD station Montabaur) [47], i.e., the elevation above sea level is a proxy of air temperature.

Aquatic habitat quality with respect to contamination by heavy metals (Zn, Pb, and Cd originating from mining waste) and by organic fertilizers (P originating mainly from local agriculture) was measured using inductively coupled plasma mass spectrometry (ICP-MS; [48]) to analyze four 100 mL water samples each collected at localities 1–8 (Figure 1).

Terrestrial habitat quality was estimated using an expert-advised ordinal rating of land use within a radius of 100 m and 500 m, respectively, around the sampling locality [49]. The ordinal scale to assess the landscape-induced mortality risk (LIMR) ranged between 1 (very low risk, e.g., forest = natural habitat) and 5 (very high risk, e.g., road with substantial car traffic). The ordinal scale to assess the landscape resistance to migration (LRM) ranged between 1 (very low resistance, e.g., leaf litter, road, trails) and 5 (very high resistance, e.g., walls, houses). The final score of LIMR and LRM was calculated as the sum of the products of single ratings for each type of landscape structure and its corresponding area divided by the total area of the circle around each sampling locality.

### 2.5. Statistical Analyses

Descriptive statistics depended on the outcome of the Shapiro–Wilks test for normality. Normally distributed data were described by the arithmetic mean and corresponding standard error or 95% confidence interval, those deviating significantly by medians and range. Correspondingly, comparisons between male and female size data were performed using t- and F-tests, those on age data using the non-parametric Mann–Whitney test.

Von Bertalanffy models were used to describe age-dependent growth [50]. Growth following metamorphosis was estimated using the equation: SVL_t_ = SVL_max_ − (SVL_max_ − SVL_met_) × e^−k×t^, where SVL_t_ = average body length at age t; SVL_max_ = asymptotic body length; SVL_met_ = body length at metamorphosis; t = number of growing seasons experienced (n LAGs), and k = growth coefficient (i.e., shape of the growth curve). The von Bertalanffy growth model was fitted to the average growth curve using the least squares procedure (nonlinear regression). Estimates of SVL_max_ and k are given with the corresponding 95% confidence interval. Sexual size dimorphism was tested based on SVL_max_. The absence of overlap between confidence intervals was considered a significant deviation at *p* < 0.05.

To compare age data among the four hydrographic systems, following a log10 transformation to fit normality, a 2-factor ANCOVA (fixed factors: sex, catchment area; continuous co-variate: altitude to distinguish between captures in the lower and the upper sampling area) and post hoc tests with Bonferroni correction were performed. Size data were analyzed in a 2-factor ANCOVA with the same fixed factors but additionally with the continuous co-variates altitude and age. To assess the contribution of potential environmental impact factors on the variance of age and size, exploratory multiple regression analyses were run on the adult datasets. The model with the lowest number of explanatory variables was chosen by applying the backward selection procedure (non-significant variables with *p* > 0.05 were successively removed). The significance level was set at alpha = 0.05. All calculations were based on the procedures of the program package STATGRAPHICS centurion for Windows, version XVIII.

## 3. Results

Skeletochronological age estimation was successful in all bone samples collected. Recently metamorphosed individuals did not have detectable LAGs, whereas juveniles had one or two LAGs. Complete resorption of the innermost LAG was never observed, as the growth period between metamorphosis and first hibernation was always at least partly visible (characteristic lamellar bone structure; Figure 2). Double lines were rarely present (in 2 out of 160 adults). The first growth zone was the broadest in almost all individuals, followed by the second growth zone that was broader than the first in a few individuals. Following the second hibernation, bone growth rate decreased in 69.8% of individuals, following the third hibernation in all. Still, a narrow growth zone between two LAGs was visible even in the oldest individuals.

### 3.1. Size at Metamorphosis and Water Quality

Heavy metal contamination of larval stream habitats varied from high (Mühlenbach, sampling site 2: 133 µg/L Zn, 0.5 µg/L Pb, 0.11 µg/L Cd) to low (Wintersbornbach: 6 µg/L Zn, 0.2 µg/L Pb, and 0.05 µg/L Cd) or to absent (Mühlenbach, sampling site 1; Griesentalbach, both sampling sites; Bienhornbach, both sampling sites). Still, heavy metal concentrations were far below the maximum levels permitted for drinking water (WHO/EU drinking water standards: 3000 µg/L Zn, 5 µg/L Pb, 3–5 µg/L Cd). Phosphorus concentration was highest in the Mühlenbach (28.3–35.0 µg/L P) and lowest in the Winterbornbach (15.4–16.8 µg/L P). Despite the differences in water quality, SVL of recently metamorphosed individuals did not differ significantly among streams (2-factor ANOVA; F_3,23_ = 1.91; *p* = 0.1619) or between the headwaters and stream mouth areas (2-factor ANOVA; F_1,23_ = 0.02; *p* = 0.8867). Average size of recently metamorphosed individuals was 32 mm (range: 27–38 mm). An exploratory multiple regression analysis (procedure: backward selection) did not identify any water contaminants as a significant factor correlating with SVL.

### 3.2. Size and Age Variation in Adults

**Figure 3:**
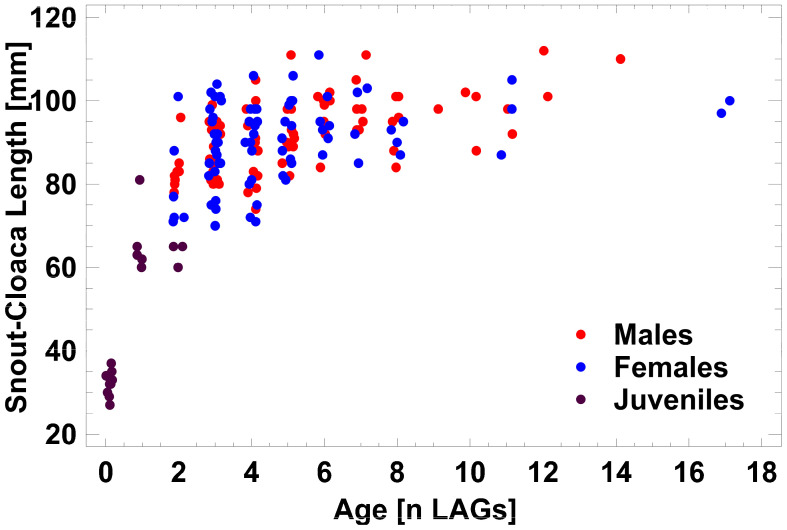
Age–size variation in 192 salamanders collected in the urban area of Koblenz. Data are pooled independent of sex for all hydrographic systems (32 recently metamorphosed individuals and juveniles, 80 males, and 80 females). Each dot represents one individual; dots are slightly jittered horizontally.

Individuals varied considerably in snout–vent length (males: 74–112 mm and females: 70–111 mm) and in age (males: 2–14 LAGs and females: 2–17 LAGs; Figure 3). SVL did not deviate from a normal distribution, whereas LAG distribution was significantly skewed. Average size did not differ significantly between males and females (92.2 ± 8.5 mm vs. 90.1 ± 10.0 mm; *t*-test: t = 1.4, *p* = 0.1704), nor did standard deviations (F_1,79_ = 0.73, *p* = 0.1568). Median age did not differ significantly either (five LAGs vs. four LAGs; Mann–Whitney test: W = 2748, *p* = 0.1179). Age-adjusted size of males and females did not differ significantly as well (ANCOVA, F_1,159_ = 0.89, *p* = 0.3421), but SVL was significantly related to age (ANCOVA, F_1,159_ = 35.9, *p* < 0.0001). The growth coefficients k of the von Bertalanffy growth models were 0.63 (CI_95%_: 0.53–0.73) in males and 0.66 (CI_95%_: 0.51–0.80) in females, the maximum extrapolated SVL 97.1 mm (CI_95%_: 94.6–99.7) in males and 95.3 mm (CI_95%_: 91.8–98.8) in females. As there was no detectable sexual size or age dimorphism, data were pooled for the further analyses. The growth model parameters based on all 192 salamanders studied were 0.66 (CI_95%_: 0.56–0.77), the maximum extrapolated SVL 96.2 mm (CI_95%_: 94.0–98.4), and variance explained by the model R^2^ = 86.2%. Most of adult SVLs were reached during the juvenile stage or in the first year of adulthood. The average SVL of young adults (88.2 mm at three LAGs) corresponded to 91.7% of the extrapolated maximum SVL. Average adult size (analysis restricted to age classes with n ≥ 3 LAGs) varied significantly between 89.1 mm (four LAGs) to 97.3 mm (seven LAGs; ANOVA; F_3,80_ = 4.29; and *p* = 0.0075), but annual size increase was small decreasing to almost zero at higher age. Maximum SVL variation (71–106 mm) was detected at the age of four LAGs.

### 3.3. Environmental Drivers of Local Demography

**Figure 4:**
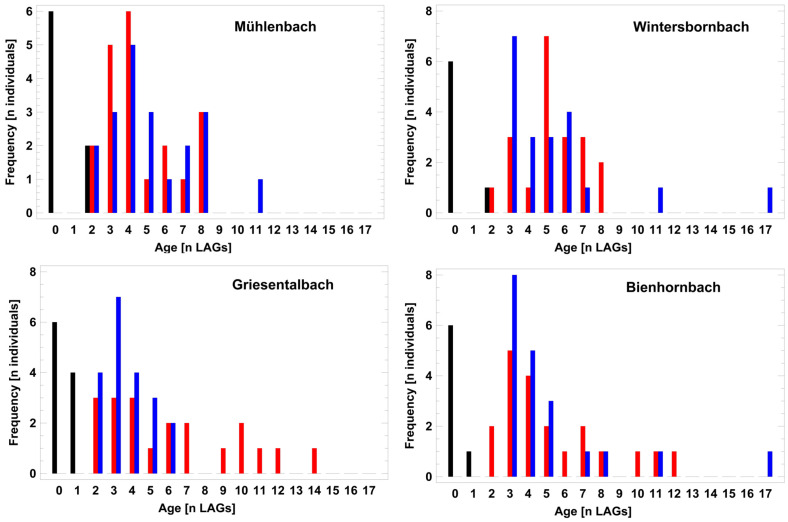
Age distribution of salamanders collected in the four catchment areas. Black bars: recently metamorphosed individuals and juveniles; red bars: males (n = 20 per catchment); blue bars: females (n = 20 per catchment).

**Figure 5:**
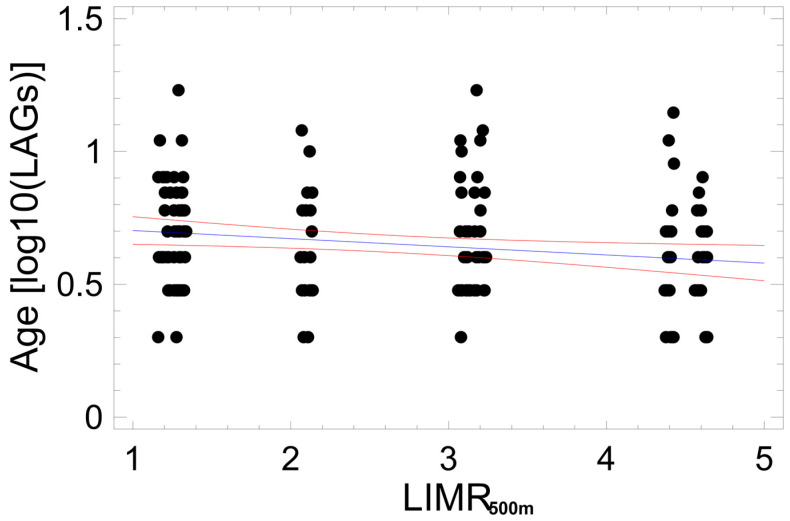
Impact of landscape-induced mortality risk within a radius of 500 m (LIMR_500m_) on the log10-normalized age of adult salamanders. Blue line = regression line, red lines = 95% confidence range. Each dot represents one individual, dots are slightly jittered. Statistical details in the text.

Log10-normalized age did not differ significantly between males and females (2-factor ANCOVA; F_1,159_ = 1.88, P = 0.1724) and among hydrographic systems (2-factor ANCOVA; F_3,159_ = 0.38, P = 0.7665). Altitude used as a co-variate to distinguish the upper and lower capture sites was unrelated to age (2-factor ANCOVA; F_1,159_ = 1.92, P = 0.1683) (Figure 4). To detect subtle, non-lethal impacts on the local demography, an exploratory multiple regression analysis (procedure: backward selection) including all recorded environmental variables was used. The only parameter significantly covarying with age was habitat quality LIMR_500m_ regarding mortality risk within a 500 m circle around the sampling area: log10(LAGs) = 0.7330 − 0.0306 × LIMR_500m_ (R^2^ = 3.6%; F_1,159_ = 5.98, *p* = 0.0156; Figure 5).

### 3.4. Environmental Drivers of Local SVL Distribution

Log10-normalized SVL differed significantly among catchment areas (2-factor ANCOVA; F_3,159_ = 10.36, *p* < 0.0001) but not between males and females (2-factor ANCOVA; F_1,159_ = 1.38, *p* = 0.2413). The co-variate altitude was not related to SVL (2-factor ANCOVA; F_1,159_ = 0.05, *p* = 0.8181), but age was (2-factor ANCOVA; F_1,159_ = 37.29, *p* < 0.0001). Salamanders inhabiting the catchment area of the Mühlenbach were significantly smaller (least squares mean: 85.3 mm vs. 91.3–94.4 mm) than those from the other three (post hoc comparison with Bonferroni correction: *p* < 0.01).

**Figure 6:**
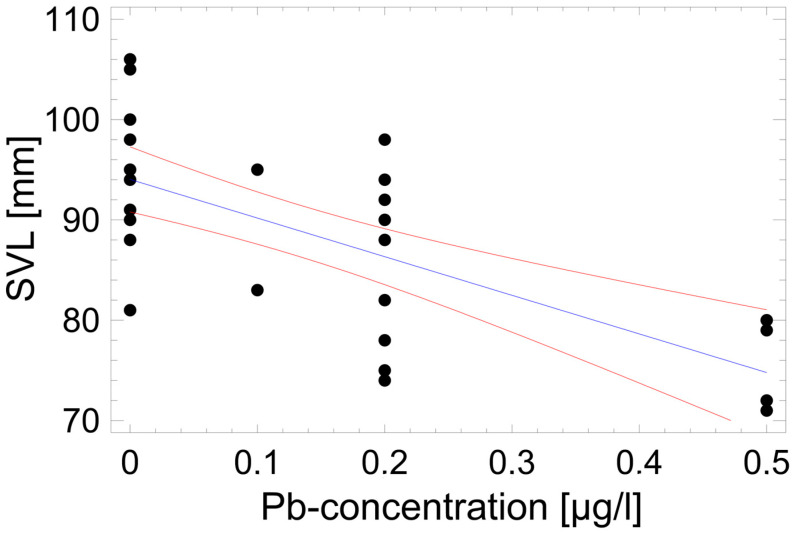
Impact of lead concentration in the stream water on the snout–vent length of salamanders of the four LAGs age class. Blue line = regression line; red lines = 95% confidence range. Each dot represents one individual. Statistical details are in the text.

As the most influential but non-linear correlate of SVL was age (see Figure 3), the analysis of environmental drivers of size variability was limited to the four LAGs age group, in which the adult SVL span was largest (35 mm between the smallest and the largest of 31 individuals). An exploratory multiple regression analysis (procedure: backward selection) identified as best fit (maximum R^2^) a model including six significant environmental impact factors: SVL = 351.8 + 3.0 × [Zn] − 168.7 × [Pb] − 2463.0 × [Cd] + 1.2 × [P] − 44.9 × LRM_100m_ − 76.9 × LIMR_500m_, explaining 62.6% of variance in SVL (F_6,30_ = 6.99, *p* = 0.0002). The Pb concentration alone accounted for 47.5% of explained variance (Figure 6), all heavy metals together for 50.7%., whereas the P concentration, the habitat quality, with respect to mortality risk (LIMR_500m_), and the landscape resistance to migratory permeability (LRM_100m_) added 11.9%.

## 4. Discussion

This study provides evidence that the local salamander populations inhabiting the four stream catchments have a similar age structure but vary considerably in adult size. The fire salamanders of the upper middle Rhine Valley mature at 2–3 years and may live up to 17 years, showing the same demographic landmarks as most conspecific populations from Poland in the north and Spain, Portugal, and Serbia in the south of the distributional range (Supplementary Table S1). Local habitat quality in terms of the mortality risk for roaming salamanders may account for small but detectable differences in local demography, first evidence for the presence of significant local scale variability. In contrast, adult size (as average SVL) varies considerably throughout the salamanders´ geographical range, with populations ranging from a minimum of ca. 85 mm (Mühlenbach, this study; Serra San Bruno, Italy [51]) over ca. 98–100 mm (Geres, Portugal; Emilia-Romana, Italy; Serbia [18,52,53]) to a maximum of ca. 117–120 mm (Despotovac, Serbia [19]), but without showing any correspondence to macroecological gradients. The combination of relatively invariable local demography associated with highly variable adult body size emphasizes that local growth rates are most probably sensitive indicators of habitat quality. This finding contrasts to those detected in newts *Triturus cristatus* and yellow-bellied toads *Bombina variegata* that show highly variable local survival rates depending more closely on habitat characteristics than size [54,55]. The presence of chemical protection increases longevity in fishes, reptiles, and amphibians [56]. This makes the difference in temperate zone anurans as well; *T. cristatus* is palatable to most predators and relatively short-lived; *B. variegata* populations differ considerably in their toxicity, whereas fire salamanders have always had high concentrations of skin toxins [54,57,58].

### 4.1. Demography

Local demography of amphibians reflects recruitment of young as well as survival of individuals [59,60,61]. Recruitment of juveniles in stream breeding salamanders is not only related to water quality but may also depend on catchment-wide riparian forest cover as a high-quality habitat [62,63]. In the four studied populations, the recruitment of first-breeders does not seem to be a limiting factor because all populations include several hundreds of adults, and the proportion of the age classes 2–3 LAGs (recently matured adults) was similar, with 30% in the most polluted stream and 38% in the cleanest. The only report on reduced survival of salamander larvae was that on a population breeding in a contaminated stream with high antimon concentrations [62,64]. In *S. salamandra,* survival in the terrestrial habitat seems to be the major driver of local demography, as evidenced in a population with constant recruitment rates that showed a long-term decline during an 18-year monitoring period [59]. The present small-scale comparison among populations supports the hypothesis of habitat quality-dependent juvenile and adult survival because the low but significant correlation (3.6%) of local longevity was exclusively related to the estimated mortality risk in the distinct catchment area (see Figure 5). The major mortality risk is being overrun by cars, motorcycles, and even bicycles on the routes crossing the migratory range since salamanders showed upon approach aggressive postures instead of escape behavior ([40,65], pers. Observations). The remarkable low variability of local demography among near and distant localities of the large geographical distribution range seems to be due to the fact that salamanders, once having reached adulthood, are protected by skin toxins against most predators.

### 4.2. Size Plasticity

The wide variation of average adult SVL among fire salamander populations throughout Europe indicates that growth rate is a more sensitive indicator of habitat quality than age. It is noteworthy that all populations studied in the upper middle Rhine Valley had small-sized adults, representing SVLs near the lower extreme of known size averages. Salamanders of the Mühlenbach population show the smallest average size ever discovered [18,19,51,52,53]. Since more than 90% of adult size is gained before attaining sexual maturity, the habitat quality of both stream and catchment area is a potential source of growth limitation.

In stream breeding populations, food availability and quality of larvae are known to influence size at metamorphosis directly and may also lead to compensatory growth during the juvenile stage, i.e., to carry-over effects decoupling aquatic and terrestrial stages ([66,67] but see [68]). In the study area, recently metamorphosed salamanders were within the SVL range recorded in other populations [19], and size did not differ among the four streams, making distinct qualities of food unlikely. Still, age-adjusted size of adults was significantly related to water quality (negative: Pb and Cd; positive: Zn and P) at the sampling areas, suggesting a coupling between the aquatic and terrestrial stages (see Figure 6). Heavy metal effects on growth and bioaccumulation in adult tissues have been observed in other amphibian taxa as well [69,70,71,72]. Lead accumulated together with other heavy metals during the aquatic stage may have had sublethal, chronic effects on growth rates at the terrestrial juvenile stage resulting in small-sized adults.

The long-term effect of heavy metal exposure and subsequent storage in the tissues was probably reinforced by additional and regular exposure of foraging salamanders to pesticides in the vineyards of the lower Mühlenbach catchment. Adverse effects of pesticides applied in viniculture have been documented [73,74,75]. The threatening combination of heavy metals during the aquatic stage and of pesticides during the terrestrial stage is the most plausible cause for the extraordinarily small size of the local adults. The salamander populations of the study area that were unaffected by these threats were significantly larger, but still much smaller than in most other localities in the distribution range [18,19,51,52,53]. Yet, terrestrial habitat quality allows for the establishment of very large salamander populations with hundreds or thousands of individuals in these widely urban habitats [40,76]. As *S. salamandra* is thought to be territorial [77,78,79], a hypothesis to be tested in the future is that the comparatively small size reflects density-dependent increased aggressive interactions with high energetic costs constraining available time for food uptake and subsequently growth rates.

## 5. Conclusions

Environmental drivers may act on local demography and body size plasticity in amphibians. In palatable species the main action is on demography with a wide local variation of survival rates, whereas in those with high concentrations of skin toxins, such as fire salamanders, survival rates vary within a narrow margin. Still, local habitat quality may account for a minor proportion of demographic variability, as demonstrated by the significant impact of landscape-induced mortality risk on the salamander populations of the upper middle Rhine Valley.

In fire salamanders, the main effect of variation in habitat quality is on body size reflecting growth rates during the terrestrial juvenile stage. Larvae exposed to non-lethal heavy metal contamination in streams may have lower growth rates during the juvenile development than those originating from streams with good water quality, providing evidence for carry-over effects from one stage to another, i.e., for environmental coupling. Yet, average body size in the four studied populations was generally rather small compared with those in other parts of the distribution range. The presence of density-dependent growth reduction in the terrestrial habitat of the middle Rhine Valley populations with hundreds and thousands of individuals still needs to be evaluated.

## Data Availability

All research data used for this study are available in Supplementary Table S2.

## Supplementary Materials

The following supporting information can be downloaded at: https://www.mdpi.com/article/10.3390/ani14192869/s1, Table S1: Literature review on male and female age at maturity and longevity in *Salamandra* spp.; Table S2: Dataset used for the analysis of *Salamandra salamandra* age and size variation. The dataset contains: the stream and sampling site in which collection of salamanders were performed together with elevation (m asl); date of the collection; sex and snout–vent length (SVL, in mm); concentrations of water contaminants at the sampling sites; rating of landscape resistance against movements and of risk of mortality.
