# Supplementary material for: Environmental Drivers of Local Demography and Size Plasticity in Fire Salamanders (Salamandra salamandra)
Source: Animals (Basel). 2024 Oct 5;14(19):2869. doi: 10.3390/ani14192869 (PMC11475656; doi:10.3390/ani14192869)
Supplement: Supplementary file 1 [file animals-14-02869-s001.zip › animals-3186883-Supplementary Table S1.pdf]

animals

Supplementary Table S1. Skeletochronogical estimates of age at maturity and maximum lifespan recorded of all currently recognized *Salamandra* spp (except for *S. corsica*).

***Salamandra* species**	**Males**		**Females**		**Locality [reference]**
	Age at maturity	Longevity	Age at maturity	Longevity	
S. salamandra	2	14	2	17	Germany [this study]
S. salamandra	2	17	2	12	Poland, Sudetes [11]
S. salamandra	2	15	2	16	Poland, Carpathians [11]
S. salamandra	3	14	5	14	Serbia [12]
S. salamandra	4	13	3	11	Portugal, Geres [13]
S. salamandra	3	18	3	16	Portugal, Sintra [13]
S. salamandra	2	13	2	11	Portugal, Monchique [13]
S. salamandra	3-4	9-12	3-4	9-12	Spain [14]
S. algira	5	20	5	19	Morocco [15,6]
S. algira	4	17	4	18	Algeria [17]
S. atra	4	15	4	15	Austria [21]
S. atra	5	10	5	11	Serbia/Slovenia [12]
S. inframaculata	3	11	4	12	Turkey [18]
S. lanzai	8	23	8	24	France, Mount Viso [19]
S. lanzai	3	22	6	22	Italy, Mount Viso [19]
S. lanzai	4	16	4	16	Italy, Germansca [20]
